# Association Between Media Dose, Ad Tagging, and Changes in Web Traffic for a National Tobacco Education Campaign: A Market-Level Longitudinal Study

**DOI:** 10.2196/jmir.5343

**Published:** 2016-02-17

**Authors:** Paul R Shafer, Kevin C Davis, Deesha Patel, Robert Rodes, Diane Beistle

**Affiliations:** ^1^ Center for Health Policy Science and Tobacco Research RTI International Research Triangle Park, NC United States; ^2^ Department of Health Policy and Management Gillings School of Global Public Health University of North Carolina at Chapel Hill Chapel Hill, NC United States; ^3^ Office on Smoking and Health Centers for Disease Control and Prevention Atlanta, GA United States

**Keywords:** Internet, advertising, health communication, smoking cessation, public health, tobacco control

## Abstract

**Background:**

In 2012, the US Centers for Disease Control and Prevention (CDC) launched *Tips From Former Smokers (Tips)*, the first federally funded national tobacco education campaign. In 2013, a follow-up *Tips* campaign aired on national cable television networks, radio, and other channels, with supporting digital advertising to drive traffic to the *Tips* campaign website.

**Objective:**

The objective of this study was to use geographic and temporal variability in 2013 *Tips* campaign television media doses and ad tagging to evaluate changes in traffic to the campaign website in response to specific doses of campaign media.

**Methods:**

Linear regression models were used to estimate the dose-response relationship between weekly market-level television gross rating points (GRPs) and weekly Web traffic to the *Tips* campaign website. This relationship was measured using unique visitors, total visits, and page views as outcomes. Ad GRP effects were estimated separately for ads tagged with the *Tips* campaign website URL and 1-800-QUIT-NOW.

**Results:**

In the average media market, an increase of 100 television GRPs per week for ads tagged with the *Tips* campaign website URL was associated with an increase of 650 unique visitors (*P*<.001), 769 total visits (*P*<.001), and 1255 total page views (*P*<.001) per week. The associations between GRPs for ads tagged with 1-800-QUIT-NOW and each Web traffic measure were also statistically significant (*P*<.001), but smaller in magnitude.

**Conclusions:**

Based on these findings, we estimate that the 16-week 2013 *Tips* television campaign generated approximately 660,000 unique visitors, 900,000 total visits, and 1,390,000 page views for the *Tips* campaign website. These findings can help campaign planners forecast the likely impact of targeted advertising efforts on consumers’ use of campaign-specific websites.

## Introduction

As websites and social media have evolved into powerful channels for tobacco advertising [1], they have also become a vital component of health education campaigns. Daily Internet use is high among US adults (82%) and most Internet users (80%) report searching for health-related information online [2]. State tobacco control programs have accelerated their creation of state-sponsored websites and social media accounts [2], offering multiple platforms to tobacco users for outreach and cessation interventions. Furthermore, Web analytics have evolved to allow organizations to assess the effectiveness of campaigns on increasing traffic to websites [3].

In 2012, the US Centers for Disease Control and Prevention (CDC) launched *Tips From Former Smokers* (*Tips*), the first federally funded national tobacco education campaign, resulting in an estimated additional 1.6 million quit attempts nationally [4]. A second wave of *Tips* aired from March 4 to June 21, 2013, using similar creative content—graphic, emotional advertisements portraying the health consequences of smoking. Approximately one-third of all television ads were tagged with the *Tips* campaign website URL [5], while two-thirds promoted the national 1-800-QUIT-NOW telephone quitline. The 2013 campaign was also supported by digital advertising, including online video, display, mobile, and paid search ads.

Previous research has shown that weekly traffic to the *Tips* website increased dramatically during the 2013 *Tips* campaign compared with the 4 weeks before and after the campaign [6]. However, a limitation of this analysis was the use of a pre-post analysis that assesses changes in Web traffic purely as a function of campaign airdates. This approach fails to account for variation in media dose during the campaign (ie, temporal variation) and across media markets (ie, geographic variation), in addition to the differences in population and sociodemographic characteristics of each market. To our knowledge, no studies have examined the relationship between varying doses of advertising for a national health education campaign and the magnitude of changes in Web traffic over time. To address this gap in the literature, we used geographic and temporal variation in 2013 *Tips* gross rating points (GRPs) to quantify market-level changes in use of the *Tips* website in response to specific unit increases in weekly dose of GRPs. These results were used to estimate the additional traffic to the *Tips* website attributable to the 2013 *Tips* television ads tagged with the *Tips* campaign website URL or 1-800-QUIT-NOW.

## Methods

### Outcome Variables

We assessed three primary outcomes of Web traffic to the *Tips* website: (1) total unique visitors, (2) total visits, and (3) total page views. These metrics were derived from Adobe SiteCatalyst, a tool that provides utilization and engagement metrics for websites.

Unique visitors included the total number of unique individuals who visited the site during a given period, regardless of number of repeat visits by each individual. Visits are defined as the total number of visits made to the website during a given time period. A visit occurs when an individual arrives at and navigates the website, even if the individual has previously visited the site. A visit may consist of multiple page views, and each individual visit continues until there are 30 minutes of browser inactivity or 12 hours of continuous activity. Page views are the total number of times that individual website pages are viewed. For example, if one user views five pages on the site, this represents five total page views for that period. For these three aforementioned outcomes, metrics for each of the 210 US media markets were aggregated for each of the 16 weeks of the 2013 *Tips* campaign and for the 4 weeks before and after the campaign—24 weeks total.

### Control Variables

The primary measure of 2013 *Tips* exposure was weekly media market-level GRPs for television ads. GRPs measure the relative “dose” of advertising delivered to a target audience in a given media market and time period. They are defined as the product of the proportion of an audience that is potentially exposed (ie, audience reach) and the frequency of that exposure (ie, number of times an ad was aired). For example, if a television ad reaches 50% of an audience twice in one week, the GRP for this ad during that week is 100 (50 x 2) [7].

During 2013 *Tips*, CDC also delivered a significantly higher dose of digital video advertising to three media markets—Cleveland, Ohio; Sacramento, California; and Tampa, Florida. This additional digital video advertising used the same creative content as the television ads, all of which were linked to the *Tips* website. To account for the impact of this higher dose of digital video advertising on Web traffic in these three markets, separately from the main effect of *Tips* television GRPs, our analysis included an indicator (fixed effect) for the higher-dose markets that is equal to 1 for those three markets and 0 otherwise.

### Statistical Analysis

We used linear regression models to estimate each outcome variable at the media market level as a function of market-level weekly television GRPs (in 100s) tagged with the *Tips* website URL and GRPs tagged with 1-800-QUIT-NOW. We estimated separate models to assess the impact of each type of ad tagging. We also controlled for week of the campaign; additional state-funded airings of *Tips* ads, measured with GRPs (in 100s); and market-level sociodemographic characteristics, including total population (in 10,000s), cigarette smoking prevalence (0-100), percentage African American (0-100), percentage Hispanic (0-100), percentage with a bachelor’s degree (0-100), and median income (in US dollars). Linear predictions were made using the observed *Tips* GRP effect and effect of higher-dose digital advertising (actual) and an alternate scenario that assumed zero television GRPs and no higher-dose digital advertising (counterfactual). The differences between the actual and counterfactual predictions for each outcome are reported as the campaign-attributable effects. All analyses were conducted using Stata 13.2 (StataCorp LP) [8].

## Results

An increase of 100 television GRPs per week for ads tagged with the *Tips* website URL was associated with increases of 650 unique visitors (*P*<.001), 769 total visits (*P*<.001), and 1255 total page views (*P*<.001) per week in each media market (see Table 1). An increase of 100 television GRPs per week for ads tagged with 1-800-QUIT-NOW was associated with increases of 280 unique visitors (*P*<.001), 334 total visits (*P*<.001), and 547 page views (*P*<.001) per week in each media market. State-funded *Tips* campaign GRPs were not associated with measures of Web traffic. Media markets that received higher-dose digital advertising had approximately 2950 more unique visitors (*P*<.001), 6050 more visits (*P*<.001), and nearly 8150 more page views (*P*<.001) at the *Tips* website per week than markets that did not receive higher-dose digital advertising. Increases in market-level smoking prevalence were significantly associated with lower weekly Web traffic. Specifically, a 1% increase in smoking prevalence was associated with a decrease of approximately 19 unique visitors, 21 visits, and 28 page views per week.

Based on these findings, we estimate that 2013 *Tips* was responsible for approximately 660,000 additional unique visitors, 900,000 additional visits, and 1,390,000 additional page views to the *Tips* website over the course of the 16-week campaign (see Table 2).

**Table 1:** Multivariate regressions for Web traffic as a function of 2013 *Tips* campaign dose.

Independent variable	*Tips* website URL, ad model coefficient (SE), *P*			1-800-QUIT-NOW, ad model coefficient (SE), *P*		
	Unique visitors	Visits	Page views	Unique visitors	Visits	Page views
Weekly GRPs^a^ for ads tagged with *Tips* campaign website URL (in 100s)	650.2 (59.8), <.001	768.5 (77.3), <.001	1254.6 (116.7), <.001	N/A^b^	N/A	N/A
Weekly GRPs for ads tagged with 1-800-QUIT-NOW (in 100s)	N/A	N/A	N/A	279.6 (27.3), <.001	333.8 (35.3), <.001	546.7 (53.3), <.001
Weekly state-funded GRPs (in 100s)	11.5 (6.0), .06	12.2 (7.8), .12	19.3 (11.8), .10	11.6 (6.0), .05	12.4 (7.8), .11	19.6 (11.8), .10
Digital advertising higher-dose markets	2951.8 (98.2), <.001	6051.3 (126.9), <.001	8173.3 (191.6), <.001	2956.1 (98.3), <.001	6056.6 (127.0), <.001	8182.0 (191.8), <.001
Population of market (in 10,000s)	-330.8 (33.3), <.001	-358.6 (43.0), <.001	-602.8 (64.9), <.001	-331.7 (33.3), <.001	-359.5 (43.0), <.001	-546.7 (53.3), <.001
Smoking prevalence of market	-19.6 (5.8), .001	-22.0 (7.5), .003	-28.7 (11.3), .01	-19.0 (5.8), .001	-21.4 (7.5), .005	-27.7 (11.3), .02
Percentage of market population that is African American	11.9 (0.9), <.001	12.8 (1.2), <.001	20.7 (1.8), <.001	12.0 (0.9), <.001	12.9 (1.2), <.001	20.8 (1.8), <.001
Percentage of market population that is Hispanic	8.9 (1.1), <.001	9.7 (1.4), <.001	15.8 (2.1), <.001	9.0 (1.1), <.001	9.7 (1.4), <.001	15.9 (2.1), <.001
Percentage of market population that has a bachelor’s degree or higher	-11.3 (3.4), .001	-12.4 (4.4), .005	-15.7 (6.7), .02	-11.1 (3.4), .001	-12.2 (4.4), .006	-15.5 (6.7), .02
Median income in market	0 (0), <.001	0.1 (0), <.001	0.1 (0), <.001	0 (0), <.001	0.1 (0), <.001	0.1 (0) <.001
Week of the campaign	-1.0 (1.7), .57	-0.2 (2.2), .91	1.1 (3.3), .74	0.2 (1.7), .92	1.1 (2.2), .61	3.3 (3.3), .31
Number of observations, n	5040	5040	5040	5040	5040	5040

^a^GRP: gross rating point.

^b^N/A: not applicable.

**Table 2:** Linear predictions of 2013 *Tips* campaign-attributable effects on unique visitors, visits, and page views for *Tips* campaign website.

Prediction scenario^a^	Unique visitors^b^, n	Visits^c^, n	Page views^d^, n
Actual (observed campaign)	1,560,000	1,850,000	2,950,000
Counterfactual (no campaign)	900,000	950,000	1,560,000
Difference (campaign-attributable effect)	660,000	900,000	1,390,000

^a^All predictions are rounded to the nearest 10,000.

^b^Unique visitors represents the number of unique users of the website over a given time period.

^c^Visits represents total number of visits (including multiples by the same individual) to the website over a given period.

^d^Page views represents the total number of pages viewed on the website [5] across all visits in a given period.

## Discussion

### Principal Findings

This is the first study to quantify specific dosing levels for television advertising that are associated with weekly traffic to the *Tips* website. Although the estimated campaign-attributable increase in visits is substantial, the increase estimated in this study—660,000 additional unique visitors—is lower than the estimated 2.8 million additional unique visitors reported in earlier aggregate results [6]. Our estimates are likely more conservative because this analysis specifically quantifies the response of website use to specific unit increases in media doses while controlling for potential confounders, as opposed to a more crude approach of comparing Web traffic during the campaign period to time periods before and after the campaign.

As expected, the impact on Web traffic of ads tagged with 1-800-QUIT-NOW was smaller than the effect of ads tagged with the *Tips* website URL. The existence of an association with 1-800-QUIT-NOW tagging suggests that *Tips* ads, regardless of tagging, may generate measurable traffic to the *Tips* website. We also found that after adjusting for market-level sociodemographic characteristics, the higher-dose digital video advertising was associated with substantial increases in Web traffic. These findings demonstrate that a targeted digital strategy can help drive consumers to online resources offered by campaigns.

Our study is limited by the relatively short period of pre- and postcampaign Web traffic data available at the media market level (4 weeks before and after) compared to the length of the campaign (16 weeks). Our estimates may also be understated because traffic from users whose market location could not be established were excluded from the analysis. We also cannot establish that visitors to the campaign website were looking for cessation information, though with data on specific pages viewed, such an analysis would be possible and could establish an important link between increases in traffic to campaign websites and information-seeking for health education.

### Conclusions

In conclusion, this analysis demonstrates a significant relationship between specific doses of television advertising and the magnitude of changes in campaign Web traffic over time. This is important given that one of the primary functions of the *Tips* campaign website is to provide smokers with information and resources to help quit; it is likely that many of the additional visitors are smokers seeking help in quitting. This study shows that direct tagging of traditional television ads with campaign website addresses and/or targeted digital advertising strategies can play a direct role in increasing the use of health-related online resources, independently of the dose-response effect of campaign intensity on Web traffic. These findings may help campaign planners forecast the likely impact of advertising efforts on consumers’ use of campaign-specific websites and optimize their campaigns to increase use of online resources.

## Abbreviations

CDC: Centers for Disease Control and Prevention
GRP: gross rating point
N/A: not applicable

## References

[1] Freeman B (2012). New media and tobacco control. Tob Control.

[2] Emery S, Aly EH, Vera L, Alexander RL (2014). Tobacco control in a changing media landscape: How tobacco control programs use the Internet. Am J Prev Med.

[3] Tian H, Brimmer DJ, Lin JS, Tumpey AJ, Reeves WC (2009). Web usage data as a means of evaluating public health messaging and outreach. J Med Internet Res.

[4] McAfee T, Davis KC, Alexander RL, Pechacek TF, Bunnell R (2013). Effect of the first federally funded US antismoking national media campaign. Lancet.

[5] Centers for Disease Control and Prevention.

[6] Centers for Disease Control and Prevention (CDC) (2013). Impact of a national tobacco education campaign on weekly numbers of quitline calls and website visitors--United States, March 4-June 23, 2013. MMWR Morb Mortal Wkly Rep.

[7] Farrelly MC, Davis KC, Nonnemaker JM, Kamyab K, Jackson C (2011). Promoting calls to a quitline: Quantifying the influence of message theme, strong negative emotions and graphic images in television advertisements. Tob Control.

[8] StataCorp (2013). Stata Statistical Software: Release 13 [Software Package].

